# Aflatoxin M_1_ in Raw Milk, Pasteurized Milk and Cottage Cheese Collected along Value Chain Actors from Three Regions of Ethiopia

**DOI:** 10.3390/toxins14040276

**Published:** 2022-04-12

**Authors:** Haftom Zebib, Dawit Abate, Ashagrie Zewdu Woldegiorgis

**Affiliations:** 1 Center for Food Science and Nutrition, College of Natural Sciences, Addis Ababa University, Addis Ababa P.O. Box 1176, Ethiopia; 2 Livestock and Fishery Core Process, Tigray Agricultural Research Institute, Mekelle P.O. Box 492, Ethiopia; 3 Department of Biology, College of Natural Sciences, Addis Ababa University, Addis Ababa P.O. Box 1176, Ethiopia; dawitabatetassew@gmail.com

**Keywords:** aflatoxin M_1_, milk, cheese, dairy value chain actors

## Abstract

Milk is a highly nutritious and perfect natural food for humans. However, when lactating animals feed on Aflatoxin B_1_ (AFB_1_)-containing feed, the hydroxyl metabolite aflatoxin M_1_ (AFM_1_) contaminates the milk and dairy products. The objective of the current study was to assess the level of AFM_1_ in raw milk, normally pasteurized milk and Ethiopian cottage cheese collected from value chain actors (producers, collectors, processors and retailers). Cross-sectional study and simple random techniques were used to collect primary samples. A total of 160 composite samples was collected; raw milk (*n* = 64), pasteurized milk (*n* = 64) and cheese (*n* = 32) was analyzed. Quantitative analysis of AFM_1_ was conducted using enzyme-linked immunosorbent assay (ELISA). The results indicate that AFM_1_ was detected in all milk products. Results along value chains show that the concentration of AFM_1_ in raw milk from collectors was significantly higher than from producers, and in pasteurized milk from processors and retailers (*p* < 0.05). However, no significant (*p* > 0.05) difference was observed in cottage cheese value-chain actors in all regions. Comparison of AFM_1_ mean values among all dairy products shows that raw milk had a significantly higher concentration of AFM_1_ followed by pasteurized milk and cottage cheese. However, there was no significant difference between raw and pasteurized milk (*p* > 0.05). The mean AFM_1_ contamination in milk products ranged from 0.137 to 0.319 µg/L (mean value 0.285 µg/L). The contamination percentages of AFM_1_ in raw milk (62.50%), pasteurized milk (67.20%) and cottage cheese (25%) were above the regulatory limit set by the European Union (EU) (0.05 µg/L). According to USA/Ethiopian Standard (US/ES) (0.50 µg/L), 21.87%, 25% and 1% exceeded the regulatory limit for the above products, respectively. The overall prevalence (56.88%) was above the EU regulatory limit and 19.38% over US/ES regulations. Therefore, to provide accurate information about the health risk to consumers, there is a need to conduct risk assessment studies in consumers of milk and dairy products at different age groups.

## 1. Introduction

Aflatoxins are natural contaminants formed as secondary metabolites by toxigenic fungi in the field and/or during storage of plant-based foods and feed [1,2] which have suitable favorable conditions for the growth of aflatoxigenic fungi in tropical climates. The main producers of aflatoxins are the toxigenic fungi *Aspergillus flavus* and *A. parasiticus* [3]. The toxins can be produced in the field prior to harvest and arise due to fungal growth under poor handling and storage conditions [4]. Aflatoxins are classified as B_1_, B_2_, G_1_, and G_2_. Exposure to excess levels of aflatoxin can result in acute aflatoxicosis, leading to jaundice, oedema, gastrointestinal hemorrhage and, ultimately, death [1,5]. However, the most common form of exposure is chronic, which contributes to the development of liver cancer.

AFB_1_ is a carcinogenic toxin that can be found in different cereals as well as contaminated feed [6]. The presence of AFB_1_ in contaminated feed is hydroxylated to the hydroxyl metabolite AFM_1_, also known as milk toxin, and the subsequent exposure of lactating animals to it leads to the contamination of milk with AFM_1_ [7,8,9]. AFM_1_ can be a potential health hazard to humans, particularly infants and children, since milk is the major constituent of their diet and thereby, they are more susceptible to its effect than adults [6,10,11].

In Ethiopia, the dairy sector is very important to nutrition, and the economy. Dairy products are consumed in rural, urban and peri-urban areas by different age groups in different regions. Raw milk supplied from urban and peri-urban producers is processed into pasteurized milk, ‘*ergo*’ (naturally sour whole fermented milk yoghurt), cottage cheese (*Ayib*) and whey (*Aguat*) [12,13].

Regulatory limits for the level of AFM_1_ residues have been established in many countries. However, Ethiopia has adopted international standards or established specifications limits in milk and dairy products without conducting comprehensive research representing the country. There are data regarding contamination of dairy products with AFM_1_ in various countries. However, there is a scarcity of research data regarding AFM_1_ occurrence in milk and dairy products in Ethiopia. So far, only two published research works [14,15] report on AFM_1_ occurrence in raw and pasteurized milk and cottage cheese in specific district areas (around Addis Ababa). Moreover, there is little information on milk products collected from the retail market in different regions. This study reflects the incidence of AFM_1_ in the country, with representative large sample sizes covering wider areas and including major value chain actors in the country. It is intended as an input for the risk assessment of milk and dairy products to consumers. Therefore, the aim of study was to assess concentration of AFM_1_ in raw milk, pasteurized milk and Ethiopian cottage cheese collected along value chain actors from three regions of Ethiopia.

## 2. Results

### 2.1. Recovery and Reproducibility Data

The recovery and reproducibility data are shown in Table 1. The recovery percentage for raw milk, pasteurized milk and cottage cheese were 95.91%, 89.37% and 89.04% respectively. Intra-assay precision for the above products within assay were 4.74%, 3.46% and 3.60% coefficient of variation (CV), respectively. The inter-assay precision for AFM_1_ free milk and 0.05 ppb standard were 11.09% and 5.12% CV, respectively. This guarantees that the results obtained throughout the study were reproducible. Method limit of detection (LOD) was 0.002 µg/L.

### 2.2. Occurrence of AFM_1_ in Raw Milk, Pasteurized Milk and Cheese along Value Chain Actors

The level of AFM_1_ in raw and pasteurized milk among value chain actors are presented in Table 2. All milk products were contaminated with AFM_1_ in all study regions. The mean AFM_1_ levels for the Oromia, SNNP and Amhara regions were 0.421, 0.30 and 0.037 µg/L respectively. The concentration of AFM_1_ in samples from collectors was significantly (*p* < 0.05) higher than that of producers and retailers in Oromia region. In Amhara region, there were no significant differences (*p* > 0.05) between the raw and pasteurized milk samples among value chain actors in terms of AFM_1_ concentration. However, we found significantly higher AFM_1_ values from producers and collectors than processors and retailers in SNNP region. In all regions, no varying AFM_1_ concentration in pasteurized milk was observed between processors and retailers. Higher mean AFM_1_ concentration (0.512 µg/L) was obtained in Oromia region, while it was lower (0.045 µg/L) in Amhara region.

The results of analysis of 32 cheese samples for AFM_1_ are shown in Table 3. All the cheese samples were found to be contaminated with AFM_1_ among value chain actors within all regions. However, statistical analysis did not show significant (*p* > 0.05) difference among value chain actors in all regions. The level of AFM_1_ was ranged from 0.014–0.0539 µg/L in all regions.

### 2.3. Variation and Comparison of AFM_1_ Level in Milk and Cheese with Regulatory Limit Value

Comparison of AFM_1_ among milk products is summarized in Table 4. In our study, AFM_1_ contamination in raw milk ranged from 0.003 to 2.177 µg/L (mean value 0.319 µg/L). Raw milk samples (62.50%) were above the regulatory limit set by the EU and 21.87% were over the US limit. Of pasteurized milk analyzed (*n* = 64), 49 samples (67.20%) exceeded the EU limit and 16 samples (25%) were above the US/ES regulatory limit, with AFM_1_ levels ranging from 0.011 to 1.798 µg/L and having a mean value of 0.324 µg/L. The obtained results also show that 67.20% and 25% of pasteurized milk collected from processors and retail markets in the country were higher than the regulatory limit as accepted by the EU and US respectively. For cottage cheese, analytical results indicate that 25% surpassed the EU maximum limit, and only 1% was over the US/ES regulatory limit.

When results were compared, raw milk had a significantly higher concentration of AFM_1_ followed by pasteurized milk and cottage cheese. However, we observed no significant (*p* > 0.05) difference between raw milk and pasteurized milk. The concentration of AFM_1_ in cottage cheese was 2.32- and 2.36-fold lower than in raw and pasteurized milk, respectively. In this work, the overall prevalence percentages were 56.88% and 19.38%, above EU and US/ES regulatory limit values, respectively, which were lower than previously reported prevalence results in the country [14,15].

## 3. Discussion

AFM_1_ contamination in milk products had varied among value chain actors. Higher AFM_1_ concentration was found in collection centers than producers. This variation could be due to large-volume mixing of different levels of AFM_1_-contaminated raw milk from different producers, through formal and informal means, at the milk collection centers, thereby increasing the level of AFM_1_ in mixed raw milk. This can, thereby, contribute to the occurrences in its processed products. In Ethiopia, 95% of milk is channeled through the informal market, where dairy farmers sell to neighbors, small unions, cooperatives and retailers [16]. A study carried out in Iran showed the concentration of AFM_1_ in raw milk sampled from dairy producers was significantly lower than that in samples from collectors and processors [17], which is similar to the finding of this work. The variation in content of AFM_1_ among regions could be due to accessibility and the use of different levels of contaminated animal feed type, resulting from poor preharvest handling, postharvest management and poor storage conditions. The use of contaminated concentrated feed in Oromia region districts was previously reported by Ref. [14]. Raw milk originating from different farmers’ management practices, wide agroecological conditions and fluctuating climate change could be the factors for disparity in AFM_1_ content [18]. Ref. [9] conducted a comprehensive study on the occurrences of AFB_1_, deoxynivalenol and zearalenone in animal feed samples collected from various regions of China in the last three years, and found that raw feed ingredients and concentrates were seriously contaminated with these toxins. They recommended regular monitoring, application of strategies for mycotoxin control and setting regulatory limits on mycotoxin co-contamination in the feeds.

The previous investigation in the country by Ref. [14] was conducted to determine AFM_1_ levels in raw milk collected from producers and collectors (overall mean 0.41, max value 4.98 µg/L). Of these, only 8.20% were under the EU regulatory limit and 26.30% exceeded the US maximum limit value. They concluded that concentrate feed contaminated with AFB_1_ and used by producers was associated with an elevated AFM_1_ content in raw milk. A similar study carried out by Ref. [15] also found that 96% and 82% liquid milk were over EU and US regulatory limits, respectively. The prevalence of the previous results was lower than the current finding. This could be due to the representative large sample size used, coupled with it covering wide potential study districts in the country. In Bangladesh, Ref. [19] observed that 78.60% of milk products (*n* = 145) were contaminated, with AFM_1_ levels ranging from 0.005 to 0.198 µg/L, which is lower than the current study results. A further study [17] investigated 55.56% contaminated raw and pasteurized milk (mean, 0.021 µg/L) collected from southern Iran. They conclude that the type of farm, location and season did not bring any influence in the concentration of AFM_1._

In Kenya, levels of AFM_1_ (26.40%) exceeded the EU limit in 97 randomly selected dairy farms. Dairy farms feeding concentrates contributed to levels exceeding 0.050 µg/L [20]. In addition, Ref. [21] observed significantly varying AFM_1_ levels in milk products (raw, pasteurized, ultra-heat-treated milk/UHT, yoghurt and *lala*) collected from retail markets. More than 50% of dairy products exceeded the EU limit, but only three samples exceeded the US regulatory limit. In these products, the geometric mean AFM_1_ level was 0.062 µg/L from low-income areas (*n* = 135), whereas in the higher income area (*n* = 156) it was 0.036 µg/L. They showed that consumers were consuming contaminated dairy products containing AFM_1_ above 0.050 µg/L. Furthermore, Ref. [18] aimed to determine the prevalence of AFM_1_ in informally marketed milk (*n* = 96) in peri-urban areas using ELISA. Two-thirds of samples had above 0.050 µg/L and 7.50% samples surpassed 0.50 µg/L. For fresh milk samples collected from the Jordanian market, 66% were higher than the EU regulatory limit and 23% were above US regulatory limit [22].

In the current study, all pasteurized milk collected from retail market (shops and supermarkets) contained different levels of AFM_1_. We observed no significant difference between raw milk and pasteurized milk, which implies the pasteurization processes did not bring significant AFM_1_ level reductions. This finding is supported by previous reports [10,23]. The investigation conducted by Ref. [11] in Lebanon found contaminated raw milk (0.011–0.440 µg/L), pasteurized and UHT milk (0.013–0.219 µg/L) and dairy products (0.015–7.35 µg/L). Ref. [17] reported presences of a high contamination rate (91.67%) of pasteurized milk, (mean 0.032 µg/L), but only 18.80% of the samples were higher than the EU regulatory limit. In Portugal, pasteurized and UHT half-skimmed milk brands collected from the market were analyzed using ELISA. Out of 40 samples, two milk samples (5%) surpassed the EU regulatory limit [24]. Pasteurized (100%) and UHT (91.30%) exceeded EU regulatory limits, while 4.50% of pasteurized milk exceeded the US/new Serbian regulation [25], which was higher than the present findings.

In the present work, variations in the AFM_1_ content of cottage cheese in producers in different regions were found. This could be due to differences in AFM_1_ content of the original raw milk in the regions, and fermentation time in destabilization of protein casein during preparation. *Ayib* is processed after churning of *ergo* by removing fat, followed by gentle boiling of buttermilk and draining off whey, which is different from the soft cottage cheese made in different parts of the world. The major step of traditional cheese preparation is that the raw milk is fermented for 2–3 days under ambient temperature to make *ergo* [26]. During this process, there is production of higher levels of lactic acid bacteria in lowering pH. This had an effect on the adsorption of AFM_1_ in yoghurt coagulum, by denaturing and coagulating casein protein.

In our study, the lower values in cottage cheese could be due to lower AFM_1_ in the original raw milk being carried over into traditionally processed cheese, and a reduction of AFM_1_ during the traditional cottage cheese-making process. Ref. [27] successfully demonstrated that a reduction of AFM_1_ levels to 57.33% in raw milk and 54.04% in sterilized milk (control) during *ergo* production. They found reduced level of AFM_1_ in fresh milk samples during fermentation to *ergo* due to the proliferation of naturally present lactic acid bacteria and a progressive reduction of pH values. Significantly decreased levels of AFM_1_ from original milk during fermentation of yoghurt might be attributed to factors such as low pH, formation of organic acids and other fermentation byproducts contributed by the function of lactic acid bacteria (*Lactobacillus bulgaricus* and *Streptococcus thermophilus*) [28,29,30,31]. The acidic pH environment leads to denaturation and coagulation of casein protein thereby having an effect on occlusion of AFM_1_ in yoghurt coagulum. Furthermore, in Kenya, fermentation of raw milk into *lala* (a traditional fermented drink) significantly reduced AFM_1_ levels (71.80%) after incubation at room temperature for 15 h, and a 73.60% reduction was also observed during yogurt preparation at 45 °C for 4 h.

However, boiling did not have any effect on the reduction of AFM_1_ [18]. In contrast, no effect of fermentation in yoghurt AFM_1_ content was obtained by Ref. [32].

In Iran, the average concentration of AFM_1_ in contaminated traditional cheese samples was found to be 0.139 µg/L in the range of 0.050–0.308 µg/L [31]. They found lower concentrations of AFM_1_ in the ripened cheeses, which could be due to the function of *Lactobacillus* and *Lactococcus* strains during ripening time. The research performed by Ref. [33] analyzed 38 cheese samples and found that only 5% were above the Turkish legal limit, with AFM_1_ levels between 0.012–0.378 µg/L. The main reasons for variations of AFM_1_ concentrations in cheese are contamination levels in the original milk, processing procedures, type of cheese, condition of ripening, analytical methods used and protein content of cheese [10,31,34,35].

## 4. Conclusions

In the present study, all milk products collected among value chain actors were contaminated with AFM_1_. A prevalence of 56.88% and 19.38% were found to be above the regulatory limits set by EU and US, respectively. The fundamental intervention for the problem is to have quality animal feed production by value chain actors (farmers, co-operatives and feed traders). Moreover, strict regulations should be implemented in feed and milk value chain actors. Therefore, to provide accurate information about health risk of consumers, there is a need to conduct risk-assessment studies with dairy consumers at different age groups.

## 5. Materials and Methods

### 5.1. Study Area

**Figure 1:**
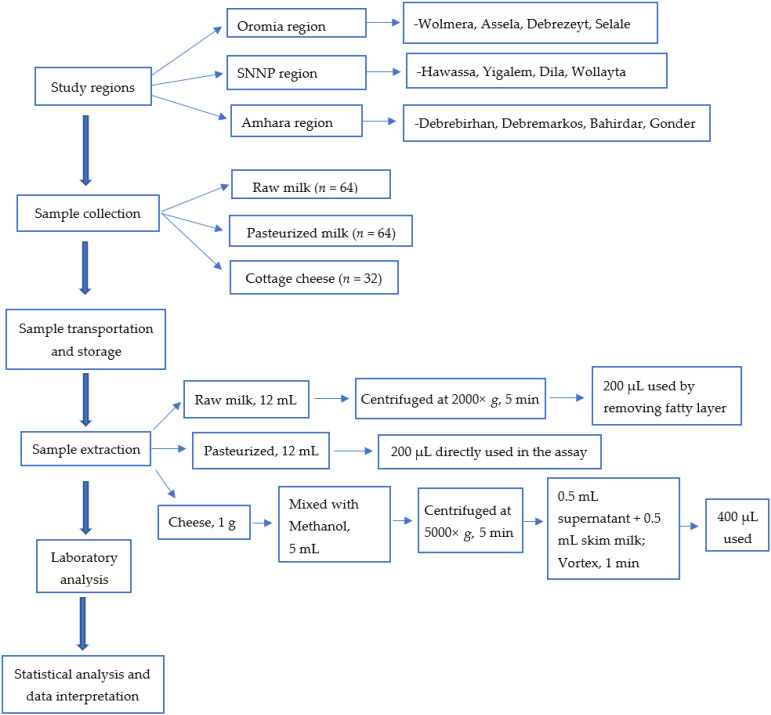
Flow diagram of research methodology.

The study areas for sample collection were selected according to Central Statistical Agency based on the location where the production of milk is high [36]. These areas include urban and peri-urban areas of Oromia, Southern Nation, Nationalities and People (SNNP), including the current Sidama region and Amhara regions, for value chains actors such as producers, collectors, processors and retailers. In each region four study areas were selected. In Oromia: Wolmera, Debrezeyt/Bishoftu, Asela and Selale; in SNNP: Hawassa, Yirgalem, Dilla and Wollayta; and in Amhara region: Debrebirhan, Debremerkos, Bahirdar and Gondar. The description of research methodology is depicted in Figure 1.

### 5.2. Laboratory Sample Collection, Transportation and Storage

All primary samples were collected from January 2019 to April 2019 in Oromia region in dry season, and from December 2019 to March 2020 in the SNNP and Amhara regions in dry season. Sampling periods were interrupted because of the COVID-19 pandemic. Laboratory raw milk samples were taken using sterilized equipment, sampling apparatus and containers according to Ref. [37]. Cow’s raw milk was sampled from producers and collectors, whereas pasteurized milk was sampled from processors and retailers. Primary samples of raw milk (about 200 mL) were collected in autoclavable, screw-capped clean plastic bottles (250 mL capacity). Pasteurized milk (500 mL capacity), processed at high temperature for a short time (72 °C, 15 s) was purchased from processors and supermarkets/shops before their expiry date. Ethiopian cottage cheese (about 200 g) was collected from producers (dairy farmers) and local retail markets in zipper polyethylene plastic bags. All samples were transported in a thermoelectric cooler and brought to the Center for Food Science and Nutrition laboratory of Addis Ababa University within 48 h of the sampling period and stored at −20 °C until analysis.

### 5.3. Sample Design, Sample Size and Composite Samples

Cross sectional study was used to collect primary samples. Producers and retailers were selected using a simple random technique, whereas collectors and processors were selected purposefully. Composite samples (*n* = 160) of raw milk (*n* = 64), pasteurized milk (*n* = 64) and cottage cheese (*n* = 32) were analyzed. This was performed by mixing 5 primary samples together by taking about 50 g per sample to obtain one representative composite sample among value chain actors (producers, collectors, processors and retailers) in each region according to [38].

### 5.4. Sample Preparation for AFM_1_ Analysis

The sample preparation procedure for all milk products was conducted according to the manufacturer’s manual [39]. All milk samples were removed from the refrigerator and allowed to stand at room temperature. An aliquot of raw milk sample (12 mL) was centrifuged at 2000× *g* for 5 min to induce separation of the upper fatty layer. The lower plasma (200 µL) was used in the assay by removing the upper fatty layer by aspiration. Normally, pasteurized milk was used directly in the assay due to the stabilization of the fat globules induced by the homogenizing process, since they are difficult to remove even by high-speed centrifugation to create a plasma from homogenized fatty milk. For cottage cheese, 1 g was mixed with 5 mL of absolute methanol in a capped tube and mixed for 5 min. The tube was clarified by centrifugation (5000× *g* for 5 min). From this, 0.5 mL supernatant was transferred to a glass tube and the methanol was evaporated by a stream of air (nitrogen gas). Semi-solid viscous material was deposited at the bottom of the tube. After that, 0.5 mL of the blank skim milk (provided with kit) was added to the tube and vortexed vigorously for 1 min. Then the tube was allowed to stand for a further 5 min and 2 × 200 µL was used in the assay.

### 5.5. Quantitative Analysis of AFM_1_ by ELISA Method

Quantitative analysis of AFM_1_ using the ELISA method is faster and more reliable, simple and cost-effective for large sample size analysis than other methods [40]. Analysis of AFM_1_ in milk and cottage cheese was conducted using the ELISA method by HELICA AFM_1_ Assay (CAT. NO. 961AFLM01M-96; Helica Biosystems Inc., Santa Ana, CA, USA), which is used for the quantitative analysis of AFM_1_ in milk and dairy products. The HELICA AFM_1_ Assay is a solid-phase competitive enzyme immunoassay. An antibody with a high affinity for AFM_1_ was coated onto polystyrene micro wells. The high sensitivity ELISA kit had AFM_1_ standard solutions at the following concentrations: 0.0, 0.005, 0.01, 0.025, 0.05 and 0.1 µg/L.

The ELISA kits were brought to room temperature before analysis. Samples and standards were directly added into the assay well. The plate was covered with sealing tape to avoid evaporation and protect it from excess UV light, and the well was incubated at room temperature for 2 h. Then, the contents of the wells were discarded into an appropriate receptacle and the wells were washed with PBS buffer solution three times. Wells were tapped face down on a layer of absorbent to remove the residual wash buffer. Then, 100 µL of the horseradish peroxidase (HRP) conjugate was added to each well, the plate was re-sealed and the well incubated at ambient temperature for 15 min. Next, the contents of the wells were poured out into an appropriate receptacle and the wells were washed with PBS buffer solution three times. Wells were tapped face down on a layer of absorbent to remove the residual wash buffer. In the next stage, 100 µL of enzyme substrate tetramethylbenzidine (TMB) was added to each well, covered to avoid direct light and incubated for 15 min. The reaction was stopped by adding 100 µL of stop solution, which changed the color from blue to yellow. The optical density (OD) of each microwell was measured with a microtiter plate reader (Bio-Tek Instruments, Inc., Winooski, VT, USA) using a 450 nm filter. The OD value of each microwell was recorded. The zero standard was stated as 100% binding (Bo), binding% (%B) was calculated for each standard and sample as a percentage of the zero binding (%B/Bo). The intensity of the color was directly proportional to the amount of bound conjugate and inversely proportional to the amount of AFM_1_ in the standard or sample. Therefore, as the concentration of AFM_1_ in the sample or standard was increased, the intensity of the blue color was decreased. Those samples, which were beyond the range of the highest standard concentration, were further diluted to reduce the absorbance within the range.

#### Results Calculation and Interpretation

The obtained results were calculated and interpreted according to the manufacturer’s manual [39]. A dose–response curve was constructed using either the unmodified OD values or the OD values expressed as a percentage of the OD of the zero standards against the AFM_1_ concentration of the standard. The concentration of unknown samples was measured by interpolation from the standard curve. The percentage of absorbance was calculated based on Equation (1). The zero standard was made equal to 100% and the absorbance values of other standards and samples were quoted in percentages of this value.

(1) $$Absorbance\;\left(\%\right)=\frac{Absorbance\;standard\;\left(sample\right)}{Absorbance\;of\;zero\;standard}\times 100$$

Equation (1). Calculation of percentage absorbance.

The values calculated for the standards were entered in a system of co-ordinates on semi-logarithmic graph paper against the AFM_1_ concentrations in μg/L. In order to measure actual AFM_1_ concentration (μg/L) in a sample, the concentration obtained from the calibration curve was further multiplied by the corresponding dilution factor. This is 1 for raw milk and pasteurized milk and 5 for cottage cheese.

### 5.6. Recovery and Reproducibility

To validate our method, a recovery in each product was carried out by spiking AFM_1_ (0.05 μg/L) standard into raw milk, pasteurized milk and cottage cheese samples. For spiking, the lowest values of raw milk (Amhara region), pasteurized milk (Oromia region) and cottage cheese (Amhara region) were used. Intra-assay precision in raw milk, pasteurized milk and cottage cheese were performed by running the same sample on one plate. Inter-assay precision was also performed by assaying AFM_1_ free milk and 0.05 µg/L standard provided with kits during the study period.

### 5.7. Statistical Analysis

All ELISA readings (Supplementary Material Spread-sheets S1) were performed in duplicate analysis and the data were expressed as mean ± SD median, and range. One-way ANOVA and multiple comparison tests were used to separate significantly different means; significance was accepted at the probability *p* < 0.05. All the analyses were carried out using Statistical Product Service Solution (SPSS) version 21 software. Descriptive statistics and Microsoft Excel were also used to summarize the data.

## Figures and Tables

**Table 1:** Recovery and reproducibility data.

Spike Recovery (%)											
Sample Type	Sample Reading		Spike Conc		Expected		Spiked Sample Reading		Recovery (%)		Average Recovery (%)
Raw milk	0.0031		0.050		0.0531		0.0512		96.46		95.91
	0.0031		0.050		0.0531		0.0506		95.35		
Pasteurized milk	0.0106		0.050		0.0606		0.0546		90.09		89.37
	0.0106		0.050		0.0606		0.0538		88.66		
Cottage cheese	0.0139		0.050		0.0639		0.0573		89.60		89.04
	0.0139		0.050		0.0639		0.0566		89.60		
Intra-assay precision											
Sample type	Assay 1		Assay 2		Assay 3		Average		CV within assay		CV within assay (%)
Raw milk	0.0059		0.0057		0.0063		0.0060		0.0474		4.74
Pasteurized milk	0.0055		0.0052		0.0055		0.0054		0.0346		3.46
Cottage cheese	0.0454		0.0455		0.0484		0.0464		0.0360		3.60
Inter assay precision											
	Assay 1	Assay 2	Assay 3	Assay 4	Assay 5	Assay 6	Assay 7	Assay 8	Average	SD	CV b/*n* assay (%)
AFM_1_ free milk	0.0026	0.0022	0.0023	0.0022	0.0021	0.0017	0.0022	0.0025	0.0022	0.0002	11.09
0.05 μg/L standard	0.0543	0.0489	0.0502	0.0528	0.0496	0.0497	0.0566	0.0543	0.0521	0.0027	5.12

Spike recovery values were duplicate analysis (*n* = 2); Intra-assay values were triplicate analysis (*n* = 3); Inter-assay precision values were 8 round assays; AFM_1_ = Aflatoxin M_1._

**Table 2:** AFM_1_ concentrations in raw and pasteurized milk among value chain actors in three regions of Ethiopia.

Study Regions	Value Chain Actors	Type of Product	*N*	Level of AFM_1_ (µg/L)				
				Min.	Max.	Mean ± SD	Median	Range
Oromia	Producers	Raw milk	16	0.004	1.313	0.348 ± 0.42 ^abc^	0.079	1.309
	Collector	Raw milk	16	0.028	2.177	0.750 ± 0.73 ^d^	0.511	2.149
	Processors	Pasteurized milk	16	0.011	1.610	0.530 ± 0.49 ^cd^	0.411	1.599
	Retailers	Pasteurized milk	16	0.031	1.681	0.421 ± 0.39 ^bc^	0.396	1.650
	Total		64	0.004	2.177	0.512 ± 0.53	0.399	2.173
SNNP	Producers	Raw milk	8	0.004	0.438	0.138 ± 0.17 ^ab^	0.079	0.434
	Collectors	Raw milk	8	0.012	0.370	0.133 ± 0.11 ^ab^	0.120	0.358
	Processors	Pasteurized milk	8	0.014	1.798	0.298 ± 0.61 ^abc^	0.076	1.784
	Retailers	Pasteurized milk	8	0.026	1.777	0.300 ± 0.60 ^abc^	0.091	1.751
	Total		32	0.004	1.798	0.217 ± 0.43	0.094	1.794
Amhara	Producers	Raw milk	8	0.003	0.100	0.035 ± 0.04 ^a^	0.023	0.097
	Collector	Raw milk	8	0.005	0.139	0.049 ± 0.05 ^ab^	0.029	0.134
	Processors	Pasteurized milk	8	0.012	0.150	0.060 ± 0.05 ^ab^	0.054	0.138
	Retailers	Pasteurized milk	8	0.024	0.067	0.037 ± 0.02 ^a^	0.027	0.043
	Total		32	0.003	0.150	0.045 ± 0.04	0.028	0.147

All values were duplicate analysis (*n* = 2); Mean values in column with different letter superscript are significantly different (*p* < 0.05); AFM_1_ = Aflatoxin M_1._

**Table 3:** AFM_1_ concentrations in cottage cheese from producers and farm markets in three regions of Ethiopia.

Study Regions	Value Chain Actor	*N*	Level of AFM_1_ (µg/L)				
			Min.	Max.	Mean ± SD	Median	Range
Oromia	Producer	8	0.020	0.478	0.087 ± 0.16	0.025	0.458
	Farm market	8	0.026	0.484	0.148 ± 0.16	0.075	0.458
Total		16	0.020	0.484	0.117 ± 0.16	0.039	0.464
SNNP	Producer	4	0.014	0.450	0.132 ± 0.21	0.032	0.436
	Farm market	4	0.016	0.027	0.022 ± 0.01	0.023	0.011
Total		8	0.014	0.450	0.077 ± 0.15	0.024	0.436
Amhara	Producer	4	0.033	0.484	0.150 ± 0.22	0.042	0.451
	Farm market	4	0.044	0.539	0.322 ± 0.22	0.351	0.494
Total		8	0.033	0.539	0.236 ± 0.22	0.151	0.506

All values are duplicate analysis (*n* = 2); AFM_1_ = Aflatoxin M_1._

**Table 4:** Level of AFM_1_ in raw milk, pasteurized milk and cottage cheese in three regions of Ethiopia.

Product Type	*N*	Level of AFM_1_ (µg/L)					*N* (<0.05 µg/L)	*N* (<0.5 µg/L)	*N* (>0.5 µg/L)	*N* (0 µg/L)	Regulatory Limit Value of AFM_1_ (µg/L) in Different Countries					
		Min.	Max.	Mean ± SD	Range	Median										
											EU/Codex (<0.05)		US FDA/ES (<0.5)		Egypt (0)	
											Under Limit (%)	Over Limit (%)	Under Limit (%)	Over Limit (%)	Under Limit (%)	Over Limit (%)
Raw milk	64	0.003	2.177	0.319 ± 0.50 ^ab^	2.174	0.084	24	50	14	0	37.50	62.50	78.13	21.88	0	64
Pasteurized milk	64	0.011	1.798	0.324 ± 0.46 ^a^	1.787	0.101	21	49	16	0	32.80	67.20	75	25	0	64
Cheese	32	0.014	0.539	0.137 ± 0.18 ^b^	0.525	0.038	24	31	1	0	75	25	96.90	3.23	0	32
Total	160	0.003	2.177	0.285 ± 0.44	2.174	0.074	69	100	31	0	43.13	56.88	80.63	19.38	0	160

All values are duplicate analysis (*n* = 2); Mean values in column with different letter superscript are significantly different (*p* < 0.05); AFM_1_ = Aflatoxin M_1_; EU/Codex = European Union/Codex Alimentarius Commission; US FDA = United States Food and Drug Administration; ES = Ethiopian Standard.

## Data Availability

The raw data of the results presented in this study are openly available in Mendeley Data at doi (http://dx.doi.org/10.17632/88rtjf79hg.1).

## Supplementary Materials

The following supporting information can be downloaded at: https://www.mdpi.com/article/10.3390/toxins14040276/s1, Spreadsheets S1: ELISA raw data set.
